# Psychopathy and Pride: Testing Lykken’s Hypothesis Regarding the Implications of Fearlessness for Prosocial and Antisocial Behavior

**DOI:** 10.3389/fpsyg.2018.00185

**Published:** 2018-02-20

**Authors:** Thomas H. Costello, Ansley Unterberger, Ashley L. Watts, Scott O. Lilienfeld

**Affiliations:** ^1^Department of Psychology, Emory University, Atlanta, GA, United States; ^2^Department of Psychology, The University of Melbourne, Melbourne, VIC, Australia

**Keywords:** psychopathy, pride, prosocial behavior, antisocial behavior, heroism, leadership, boldness

## Abstract

Despite widespread assumptions that psychopathy is associated with serious and repeated law-breaking, individuals with psychopathic personality traits do not invariably become chronic criminal offenders. As a partial explanation for this finding, Lykken (1995) ventured that a fearless temperament underlies both psychopathic traits and heroic behavior, and that heroic individuals’ early exposure to effective socializing forces such as warm parenting or healthy self-esteem often fosters a characteristic adaption that tends to beget “successful” behaviors, thereby differentiating heroes from convicts. In this study, we investigate relations between psychopathy, principally its fearless dominance dimension, pride, and prosocial and antisocial behavior in a community sample (*N* = 339). Fearless dominance and self-centered impulsivity components of psychopathy yielded differential relations with authentic and hubristic pride (Tracy and Robins, 2004), such that fearless dominance was significantly positively correlated with both facets of pride while self-centered Impulsivity was significantly negatively correlated with authentic pride and significantly positively correlated with hubristic pride. Further, authentic pride moderated (potentiated) the relation between fearless dominance and transformational leadership, one of the two outcome measures for prosocial behavior employed in our investigation. Authentic pride did not moderate the relations between fearless dominance and either our other measure of prosocial behavior (heroism) or antisocial behavior, nor did positive parenting moderate the relations between psychopathy components and social behavior. Unexpectedly, hubristic pride significantly moderated the relation between impulsive-antisocial features and antisocial behavior in a protective manner.

## Introduction

Cleckley’s (1941, 1976) *Mask of Sanity* famously described the heart of psychopathic personality as an enigmatic constellation of traits which entail both the outward appearance of healthy functioning, even charm—including social influence and stress immunity—and, paradoxically, brazen maladaptive or antisocial behavior. Individuals marked by psychopathic features are thought to occupy positions on every rung of the socioeconomic ladder—from business leaders and wartime heroes to smooth-talking con artists and chronic criminal offenders. Clinical lore, along with the writings of Cleckley and other prominent authors (e.g., Lykken, 1995; Patrick, 2006; Fowles and Dindo, 2009), is consistent with the possibility that psychopathy often comprises features that are largely adaptive, at least in the short term, such as fearlessness, venturesomeness, social dominance, and immunity to anxiety. Such traits may be tied to successful interpersonal behaviors and therefore bear important implications for prosocial functioning (e.g., heroism or organizational leadership) (Lilienfeld et al., 2015). In contrast, other scholars assert that adaptive traits are not relevant to psychopathy and should at best be viewed as ancillary features (Lynam and Miller, 2012). Yet for reasons that remain poorly understood, certain highly psychopathic individuals commit few overt antisocial or criminal behaviors, and a few may even lead heroic, accomplished and/or professionally rewarding lives (Smith et al., 2013; Lilienfeld et al., 2015).

Heroism, particularly, may seem a puzzling bedfellow for psychopathic personality. Regardless of how one defines heroism, it reflects—at least superficially—a form of prosocial behavior marked by risk to self. Heroic individuals are among the most revered, fabled, and enduring figures across cultures and throughout history (Campbell, 1949/2008); psychopaths, on the other hand, are commonly perceived as monstrous (e.g., Dr. Hannibal Lecter) or deceptively dangerous (e.g., Ted Bundy) criminals, even killers (but see Lilienfeld and Arkowitz, 2007/2008 and Skeem et al., 2011, for more nuanced and empirically grounded perspectives). Nevertheless, some authors (e.g., Lykken, 1995) posit that the boundary between courageous hero and callous psychopath may be more indistinct than it immediately appears. We investigate this possibility in the current work.

### Psychopathy

Researchers have conceptualized and operationalized psychopathy using several competing theoretical models, most of which can claim a modest degree of empirical support. One such conceptualization, Lykken’s (1957, 1995) seminal low fear model, views fearlessness as both the source trait (Cattell, 1973) underpinning psychopathy and its core mechanism. Moreover, Lykken theorized that fearlessness gives rise to such behaviors as interpersonal dominance, risk-taking, and persuasiveness, which, in turn, can be manifested in either socially praiseworthy (e.g., daring acts of heroism) or socially proscribed (e.g., criminality) behaviors (or both) as a function of moderator variables like warm parenting or effective socialization.

In a corporate setting, for example, fearlessness may facilitate an individual’s capacity to curry favor with or manipulate colleagues, which may foster his or her achievement of influential leadership positions (Hall and Benning, 2006). Some research bears out this conjecture, suggesting that psychopathic individuals tend to exhibit an adaptive style of leadership, termed transformational leadership (Board and Fritzon, 2005; Neo et al., 2016), wherein charismatic leaders provide vision, motivation, and guidance to followers.

Similarly, the impulsivity and grandiose narcissism associated with psychopathic personality, when paired with fearlessness, may make psychopathic individuals more likely to charge an enemy on the battlefield, fight off a mugger, make a daring escape from wartime imprisonment, and/or any number of comparable acts of heroism. Fearlessness-related traits, including a facility for steadfastly meeting intimidating challenges; comfort in a leadership role; and risk-taking proclivities, are conceptually related to heroism, and a handful of studies offer preliminary evidence that, in certain contexts, fearlessness is associated with heroic behavior (e.g., Smith et al., 2013; see also Murphy et al., 2017). In United States Presidents, for example, fearlessness appears to be tied to previous acts of wartime heroism, presidential performance, crisis management, persuasiveness, and positive relationships with congress (Lilienfeld et al., 2012). Although the arc of psychopathy does not necessarily bend toward heroism—certainly, not all psychopathic individuals are heroic—Lykken’s (1995, p. 118) famous hypothesis that heroes and psychopaths are “twigs in the same genetic branch,” itself an outcropping of the low fear model, remains an intriguing speculation, albeit one in need of corroboration.

Still, some research does present challenges to the comprehensiveness of Lykken’s low fear model, as the effect sizes linking low fear to laboratory deficits appear to be small (Hoppenbrouwers et al., 2016). Modern extensions of the theory submit that low fear is most closely tied to the development of a construct termed boldness, which reflects one of three core constructs that comprise psychopathy (Patrick et al., 2009). In their prominent triarchic model, Patrick and colleagues proposed that psychopathic traits map onto three dimensions: Boldness, Disinhibition, and Meanness. Boldness comprises social potency, insensitivity to threat, and emotional resilience. Disinhibition comprises impulsivity, interpersonal aggression, hostile attribution bias, and emotional dysregulation, and Meanness is marked by callousness, vindictiveness, and antagonism.

An allied three-factor conceptualization, typically assessed using the self-report Psychopathic Personality Inventory-Revised (PPI-R; Lilienfeld et al., 2015), describes similar separable, higher-order dimensions, Fearless Dominance; Self-centered Impulsivity; and Coldheartedness. Fearless Dominance is conceptually related to Boldness, and describes superficial charm, attenuated anxiety, and fearlessness; Self-centered Impulsivity is conceptually related to Disinhibition, and describes poor impulse control, irresponsibility, and egotism; finally, Coldheartedness is conceptually related to Meanness (although it is less saturated with antagonism than is Meanness) and consists of one subscale that captures callousness, attenuated empathy and interpersonal intimacy, and lack of guilt.

#### Risk and Protective Factors for Antisocial Behavior

The search for risk and protective factors for antisocial and criminal behavior among psychopathic individuals may help researchers to better pinpoint subgroups of psychopathic individuals who are at greater versus lesser risk for antisocial and criminal behavior, and, perhaps, ultimately target intervention efforts toward high-risk subgroups. Even among children with markedly elevated levels of callous and unemotional traits, which are believed by numerous scholars to be precursors of the core affective deficits of psychopathy (e.g., lack of empathy, lack of guilt), many do not go on to develop later conduct disorder (Frick et al., 2014), suggesting that a better understanding of protective factors is critical. Consequently, Lykken’s (1982, 1995) low fear model, which entails moderating variables (e.g., warm parenting) that foster largely adaptive (Lilienfeld et al., 2015)—or at least less maladaptive—behavioral tendencies in psychopathic individuals who do not respond well to more typical, punishment-based social reinforcers, may have far-reaching implications. With the exception of parenting, the nature and/or efficacy of such moderating variables and any attendant mechanisms of change remain(s) largely unexplored.

#### Positive Parenting

Many scholars have argued that successful socialization stems from a conjunction of temperamental characteristics and parental practices relating to the inculcation of internalized values and rules in children (Kochanska, 1993; Lahey et al., 2008; Grusec and Hastings, 2014). However, given their temperamental disposition, children with marked levels of the affective traits of psychopathy appear to be less influenced than other children by parenting practices that rely on guilt, shame, or punishment (Kochanska, 1995, 1997; Frick et al., 2003; Edens et al., 2008; but see O’Connor et al., 2016). In two longitudinal studies, Kochanska et al. (2007) found that fearless children do not tend to respond constructively to parental discipline or other forms of coercive negative feedback but, instead, may be more efficiently socialized via pathways that capitalize on positive parent-child relationships, such as consistent reinforcement for prosocial behavior. Further, CU traits tend to moderate the relation between (a) low parental warmth and ODD/CD in girls aged 7–8, such that among children with high levels of these traits, increased warmth is associated with fewer features of ODD/CD (Kroneman et al., 2011) and (b) parenting warmth and antisocial behavior in boys aged 4–12, such that increased warmth is associated with lessened anti-sociality (Pasalich et al., 2011).

#### Pride

One individual difference variable that has received no explicit research attention as a potential protective factor among individuals predisposed to psychopathy is pride. Indeed, from at least as early as Freud (1923/2001), who argued that the conscience consists of both the superego, which punishes us with guilt for inappropriate behavior, and the ego-ideal, which reward us with pride for appropriate behavior, scholars have noted that pride may serve as an alternative avenue to guilt in engendering socialization. Observing the paucity of moral emotions such as guilt and shame in psychopathic individuals, Lykken (1995) reasoned that a healthy sense of pride might function similarly in psychopaths. Specifically, psychopathic individuals imbued with healthy pride may consequently wish to maintain a conception of themselves as good and competent people, thereby predisposing them to channel their propensities into largely adaptive, or at least less maladaptive, behavioral avenues. Pride, then, may be an important and unappreciated alternative, inhibitory pathway to socialization among individuals who are largely devoid of guilt. Particularly, Lykken (1995) posited that such a prideful self-concept—and attendant view of oneself as a good, worthy, and competent person—largely stems from warm, rewarding parenting. In other words, healthy pride, in conjunction with fearlessness, is the engine driving prosocial behavior in psychopathic individuals, while positive parenting is both the ignition key and roadmap—fostering a positive self-concept, kindling pridefulness, and familiarizing pre-psychopathic children with social norms pertaining to virtuous, praiseworthy, behavior (ultimately providing those who are incentivized to maintain their healthy self-esteem with a schematic to strive toward).

### The Prideful Psychopath: Hero or Villain?

Thus, a subset of individuals with pre-psychopathic or psychopathic traits (fearlessness, in particular) who were raised in positive and well-structured environments may—by way of healthy pride—be insulated from their dispositional vulnerability to antisocial behavior, and even shepherded toward transformational leadership, acts of heroism, and/or other prosocial behavior.

#### Authentic and Hubristic Pride

Notably, philosophers and theologians have long discussed a potential fine line distinguishing adaptive from maladaptive pride, the latter often conceptualized as hubris or vanity (e.g., Hume, 1888, 2003; Damian and Robins, 2013). Tracy and Robins (2007)’s prominent account adopts just such a dichotomy, positing that pride comprises two ostensibly separable facets—authentic and hubristic—that are associated with differential behavioral and cognitive correlates (Tracy and Robins, 2007; Carver et al., 2010). Authentic pride is achievement-oriented (e.g., “I made the team because I practiced”) and largely adaptive; further, it is contingent on continuing success and effort (Tracy et al., 2009). Hubristic pride, in contrast, is rooted in grandiose narcissism, and is related to enduring beliefs about oneself (e.g., “I made the team because I am talented”; Tracy and Robins, 2007). Pride, per this dual conceptualization, is related to both adaptive (through authentic pride) and maladaptive (through hubristic pride) social and behavioral outcomes (Carver et al., 2010; Liu et al., 2016).

In this investigation, we integrate Lykken’s low fear model with the authentic/hubristic model of pride, positing that it is not merely the presence of pride *per se* that protects against antisociality and fosters prosociality in psychopathic individuals. Rather, the kind of pride that one experiences may, too, play a pivotal role in guiding social behavior. Our expectation is that fearlessness and authentic pride may be a “recipe” for everyday heroism, whereas that very same fearlessness, when instead paired with hubristic pride, often makes for villainous behavior.

### Current Study

Lykken (1995) contended that the relation between certain psychopathic features and either prosocial or antisocial behaviors may be moderated by other variables, such as healthy pride or warm parenting, which may facilitate alternative avenues to socialization in psychopathic individuals—who are often high in narcissism and deficient in fear and guilt, and thereby less averse to violating social norms than are others. Researchers have yet to test the crucial element of Lykken’s theory. As such, the current investigation assesses relations among pride, psychopathic traits, parenting style, guilt, personality, and prosocial (i.e., heroism and organizational leadership) and antisocial behaviors. Particularly, we examine whether the two “flavors” of pride outlined by (Tracy and Robins, 2007; Tracy et al., 2009) statistically influence the behavioral manifestations of psychopathic traits, and contend that pride may offer a partial explanation for the sharp divergence in outcomes among psychopathic individuals.

Specifically, we predicted that psychopathy, especially its Fearless Dominance component, will be tied to (a) decreased antisocial and criminal behaviors in the presence of authentic pride; (b) increased heroic and adaptive leadership behaviors in the presence of authentic pride; (c) increased antisocial and criminal behaviors in the presence of hubristic pride; and (d) decreased heroic and leadership behaviors in the presence of hubristic pride. Consistent with Lykken’s (1995) model, our primary interactional analyses focused on Fearless Dominance, with secondary analyses focusing on the other psychopathy dimensions of Self-centered Impulsivity and Coldheartedness. We also conducted subsidiary exploratory analyses on the PPI-R Fearlessness subscale given that it is a relatively “pure” measure of low fear.

We further hypothesized that a history of positive parenting would, like authentic pride, attenuate the association between psychopathy, especially Fearless Dominance, and antisocial behaviors, and potentiate the association between psychopathy, especially Fearless Dominance, and heroic and/or transformational leadership behaviors. We also conducted exploratory analyses examining statistical interactions with the other PPI-R dimensions.

Lastly, although our primary hypotheses focused on moderation, we were also interested secondarily in the zero-order correlations between psychopathy subdimensions, on the one hand, and heroism, leadership, criminal and antisocial behavior, parenting, pride, guilt, and narcissism, on the other. We also present correlations with narcissism to examine the specificity of our findings to psychopathy. Our correlational analyses were exploratory, with one notable exception; we predicted that positive parenting would be related to authentic, but not hubristic, pride.

## Materials and Methods

### Participants and Procedure

Participants (*N* = 339) were United States community members who responded to an advertisement posted on Amazon’s Mechanical Turk (MTurk), an online marketplace for crowdsourced labor. The battery took approximately 60 min on average to complete, and participants were compensated $3.50. Previous investigations have suggested that MTurk is an adequate source of self-report data for psychological research, providing data that are largely of equal or better quality than those provided by undergraduate samples (Buhrmester et al., 2011; Miller et al., 2017). Participants were predominantly women (56% female) and of Caucasian descent (77.7%), with a mean age of 38.6 years (*SD* = 11.4).

### Measures

#### Normal and Abnormal Personality

Participants completed several widely used measures of psychopathic and narcissistic traits, as well as general personality traits. Participants first completed the *Psychopathic Personality Inventory-Revised* (PPI-R; Lilienfeld et al., 2005) and the *Levenson Self-report Psychopathy Scale* (LSRP; Levenson et al., 1995). The PPI-R is a 154-item self-report questionnaire that yields a total score and eight lower-order subscale scores; these subscales, with the exception of PPI-R Coldheartedness (PPI-R C; α = 0.88), coalesce into two higher-order factors, PPI-R Fearless Dominance (PPI-R FD; α = 0.94) and PPI-R Self-centered Impulsivity (PPI-R SCI; α = 0.93). PPI-R Fearless Dominance comprises the subscales of PPI-R Social Influence, PPI-R Stress Immunity, and PPI-R Fearlessness; PPI-R Self-centered Impulsivity comprises the subscales of PPI-R Carefree Non-planfulness, PPI-R Rebellious Non-conformity, PPI-R Blame Externalization, and PPI-R Machiavellian Egocentricity. The LSRP is a 26-item self-report measure designed for non-institutionalized samples, yielding a total psychopathy score and two higher-order factor scores that describe primary (F1; α = 0.92) and secondary (F2; α = 0.78) psychopathy. Diverging from the PPI-R in factor structure, LSRP F1 measures self-centeredness, coldheartedness, and callousness (e.g., “Success is based on survival of the fittest; I am not concerned about the losers”), whereas F2 measures disinhibition and antagonism, along with other maladaptive traits (e.g., “When I get frustrated, I often ‘let off steam’ by blowing my top”) (Patrick et al., 2009).

We assessed narcissism with the *Narcissistic Personality Inventory* (NPI; Raskin and Terry, 1988), a 40-item self-report measure of trait narcissism from which three broad dimensions can be derived (Corry et al., 2008; Ackerman et al., 2011): Leadership/Authority (L/A; 10 items; α = 0.85), which is characterized by self-assuredness, appetite for power, and dominance (e.g., “I have a natural talent for influencing people”); Grandiose Exhibitionism (GE; 10 items; α = 0.82), which is characterized by social potency, extraversion, and drive (e.g., “I know that I am good because everybody keeps telling me so”); and Entitlement/Exploitativeness (E/E; 4 items; α = 0.44), which is characterized by self-interest, manipulativeness, and neuroticism (e.g., “I find it easy to manipulate people”). Although NPI E/E manifested a low internal consistency, this finding is consistent with Ackerman et al. (2011), who noted that the small alpha is likely due to the low number of items. Average inter-item correlation coefficient for the subscale was.17.

Participants also completed the *HEXACO Personality Inventory* (HEXACO; Lee and Ashton, 2004), a 100-item measure of dimensional personality; the HEXACO consists of 6 factors (the latter five of which correspond broadly to those in the familiar five-factor model of personality): Honesty-Humility (e.g., “I would never accept a bribe, even if it were very large”), Emotionality (e.g., “I sometimes can’t help worrying about little things”), Extraversion (e.g., “In social situations, I’m usually the one who makes the first move”), Agreeableness (e.g., “I rarely hold a grudge, even against people who have badly wronged me”), Conscientiousness (e.g., “I plan ahead and organize things, to avoid scrambling at the last minute”), and Openness to Experience (e.g., “I like people who have unconventional views”) (αs ranged from 0.83 to 0.92).

#### Potential Moderators

##### Pride and guilt

We assessed pride by means of two well-validated self-report measures: the *7-Item Authentic and Hubristic Pride Scales* (AHPS; Tracy and Robins, 2007), and the *Dispositional Positive Emotions Scale* (DPES; Shiota et al., 2006). We also examined self-esteem as a subsidiary indicator of authentic pride using the *Rosenberg Self-Esteem Scale* (RSES; Rosenberg, 1965). Self-esteem is conceptually related to authentic pride, just as grandiose narcissism is conceptually related to hubristic pride; notably, previous research and theory have suggested that, though authentic pride may be the affective “core” of genuine self-esteem (Tracy et al., 2009), the two constructs are not wholly equivalent. Further, the *Guilt and Shame Proneness Scale* (GASP; Cohen et al., 2011), a 40-item self-report measure, was administered to assess participant’s propensity for guilt and shame.

The AHPS contains two 7-item scales, each prompting participants to rate the extent to which a series of words or phrases describes them, assessing authentic (α = 0.94; e.g., “Accomplished”) and hubristic (α = 0.93; e.g., “Arrogant”) pride, respectively. Previous findings indicate that the two pride scales are generally unrelated to one another, and differentially predict theoretically relevant variables, such as narcissism, healthy self-esteem, and authenticity (Tracy and Robins, 2007; Tracy et al., 2009). However, Holbrook et al. (2014a) raised several concerns with the AHPS’ construct validity, positing that AHPS Authentic Pride captures both effort-oriented (i.e., adaptive) and ability-oriented (i.e., maladaptive) pride-related variance, whereas AHPS Hubristic Pride measures the perception that the reporter’s pride is excessive or unfounded. The DPES is a 5-item measure of positive emotionality and self-compassion (e.g., “I am proud of myself and my accomplishments”), and yields a composite score (α = 0.92), and the RSES is a 10-item self-report measure of global self-worth (e.g., “On the whole, I am satisfied with myself”), and assesses both positive and negative beliefs about the self (α = 0.94).

The GASP comprises 4 subscales. Two describe guilt, (1) Guilt-Negative Behavior Evaluation (Guilt-NBE; e.g., “After realizing you have received too much change at a store, you decide to keep it because the salesclerk doesn’t notice. What is the likelihood that you would feel uncomfortable about keeping the money?”), assessing one’s tendency to feel poorly about prior malfeasant behavior; and (2) Guilt-Repair (e.g., “You reveal a friend’s secret, but your friend never finds out. What is the likelihood that your failure to keep the secret would lead you to exert extra effort to keep secrets in the future?”), which refers to a propensity for attempts to correct past transgressions, and two describe shame, (1) Shame-Negative Self-Evaluation (Shame-NSE; e.g., “You rip an article out of a journal in the library and take it with you. Your teacher discovers what you did and tells the librarian and your entire class. What is the likelihood that this would make you feel like a bad person?”), composed of items describing negative beliefs about the self; and (2) Shame-Withdrawal (e.g., “You take office supplies home for personal use and are caught by your boss. What is the likelihood that this would lead you to quit your job?”), which measures one’s tendency to withdraw or hide after making a mistake (αs ranged from 0.67 to 0.81).

GASP subscales describing guilt proneness tend to correlate positively with measures of prosocial behavior and correlate negatively with measures of antisocial behavior, whereas shame subscales tend to correlate negatively with self-esteem and emotional stability (Cohen et al., 2011). GASP Shame-NSE and GASP Shame-withdraw often manifest differential patterns of correlations, such that individuals high in shame-NSE behave more prosocially, whereas individuals high in shame-withdraw tend to behave more antisocially.

##### Positive parenting

Parenting style was measured using a modified version of the *Alabama Parenting Questionnaire* (APQ; Frick, 1991; Essau et al., 2006), wherein participants are asked to reflect on their parents’ behavior during childhood and adolescence. Four items from the original scale were omitted to improve model fit (Essau et al., 2006); 38 items remained. In view of our hypotheses, only those items comprising the Positive Parenting subscale (α = 0.80; e.g., “Your parents told you that they liked it when you helped out around the house”) were analyzed. The APQ is often employed as a measure of parenting styles that may be related to antisociality (Essau et al., 2006), and exhibits good criterion-related validity with a bevy of conduct problems, including in clinic-referred children (Blader, 2004), non-referred children (Frick et al., 2003), and adolescents (Frick et al., 1999).

##### Antisocial and prosocial behavior

Participants completed two self-report measures assessing adaptive, successful, or socially sanctioned behaviors as proxies for prosociality—the *Multifactor Leadership Questionnaire* (MLQ; Avolio and Bass, 1995) and the *Activity Frequency Inventory* (AFI; Lilienfeld, 1998, Unpublished). Antisocial behavior was measured with the *Criminal and Analogous Behavior Scale* (CAB; Lynam et al., 1999).

The MLQ is a 26 item self-report measure of leadership style that yields a total score and scores on 3 subscales: (1) Transformational Leadership (α = 0.86; e.g., “I go beyond self-interest for the good of the group”), referring to a leader’s ability to inspire and motivate employees; (2) Transactional Leadership (α = 0.66; e.g., “I discuss in specific terms who is responsible for achieving performance targets”), also known as managerial leadership, in which leaders motivate workers based on contingent reward and punishments; and (3) Laissez-faire Leadership (α = 0.61; e.g., “I wait for things to go wrong before taking action”), wherein leaders offer little feedback or support (Jones and Rudd, 2008). Subscales possess adequate reliability and construct validity (Avolio et al., 2004).

The AFI (α = 0.86) consists of 30 items that assess lifetime performance and frequency of reasonably common heroic acts, such as attempting to break up a physical fight or helping a stranger who is in emotional distress (Patrick et al., 2006). Because acts of extreme heroism are rare, the AFI measures multiple acts of “everyday” heroism. In undergraduate and community samples, the AFI has demonstrated moderate positive correlations with both Rushton et al. (1981)’s Self-Report Altruism Scale and psychopathic personality traits; of these, the AFI appears most related to Fearless Dominance (Smith et al., 2013).

The CAB consists of 69-items assessing for frequency of engaging in various externalizing behaviors, including but not limited to alcohol and drug use, otherwise criminal behavior (e.g., driving under the influence, burglary), risky sexual behavior, intimate partner violence, and gambling. All CAB items were standardized and summed into a single composite of global antisocial behavior (α = 0.87).

## Results

### Psychopathy’s Relations with Pride, Guilt, Positive Parenting, and Social Behavior

#### Pride

AHPS Authentic Pride, RSES total scores, and DPES total scores were positively correlated with PPI-R Fearless Dominance (*r*s ranged from 0.56 to 0.69; see **Table 1**) and negatively correlated with PPI-R Self-centered Impulsivity (*r*s ranged from -0.30 to -0.44; see **Table 1**). Of PPI-R Fearless Dominance subscales, relations were significantly more pronounced for PPI-R Social Influence (*r* = 0.64) and PPI-R Stress Immunity (*r* = 0.60) than for PPI-R Fearlessness (*r* = 0.13) (tested by means of tests of dependent correlations; respectively, Steiger’s Zs were 9.39, *p* < 0.001 and 7.71, *p* < 0.001 for AHPS Authentic Pride, 9.58, *p* < 0.001 and 7.74, *p* < 0.001 for DPES total scores, and 8.53, *p* < 0.001 and 9.40, *p* < 0.001 for RSES total scores).

**Table 1:** Correlations between pride, guilt, personality features, and prosocial and antisocial behaviors.

	*M* (*SD*)	1	2	3	4	5	6	7	8	9	10	11	12	13	14	15	16	17	18	19
(1) PPI-R FD	101.78 (22.39)	—																		
(2) PPI-R SCI	33.23 (8.73)	*0.12*	**—**																	
(3) PPI-R CH	33.27 (8.39)	**0.24**	**0.35**	—																
(4) LSRP F1	28.50 (9.48)	**0.25**	**0.62**	**0.56**	—															
(5) LSRP F2	18.42 (4.99)	0.13	**0.71**	**0.18**	**0.56**	—														
(6) AHPS AP	20.47 (6.55)	**0.62**	**-0.32**	0.02	0.04	**0.47**	—													
(7) AHPS HP	9.17 (4.25)	**0.21**	**0.42**	**0.20**	**0.34**	**0.27**	0.10	—												
(8) DPES	4.87 (1.42)	**0.69**	**-0.30**	0.03	0.09	**0.42**	**0.84**	0.03	—											
(9) RSES	29.85 (7.05)	**0.56**	**-0.40**	0.01	**0.15**	**0.46**	**0.80**	*0.12*	**0.85**	—										
(10) GASP GR	5.72 (1.14)	**0.16**	**-0.56**	**0.50**	**0.53**	**0.43**	0.07	**0.27**	*0.11*	*0.13*	—									
(11) GASP NSE	5.57 (1.31)	**0.42**	**-0.35**	**0.50**	**0.47**	**0.22**	**0.18**	**0.21**	**0.15**	**0.18**	**0.52**	—								
(12) GASP SW	2.99 (1.29)	**0.27**	**0.35**	0.09	**0.25**	**0.36**	**0.36**	0.11	**0.35**	**0.39**	**0.24**	0.04	—							
(13) GASP NBE	5.33 (1.42)	**0.24**	**-0.53**	**0.56**	**0.61**	**0.34**	0.01	**0.23**	0.05	0.06	**0.64**	**0.72**	*0.14*	—						
(14) APQ	12.61 (5.31)	**0.20**	**-0.25**	**0.21**	**0.16**	**0.29**	**0.40**	0.08	**0.40**	**0.37**	**0.22**	0.09	**0.20**	**0.16**	—					
(15) CAB	0.11 (3.71)	**0.26**	**0.26**	0.03	**0.19**	**0.19**	0.05	0.03	0.07	0.04	-0.07	*-0.13*	0.05	*0.14*	-0.05	—				
(16) TF	18.81 (3.08)	**0.28**	**-0.38**	**0.39**	**0.37**	**0.43**	**0.46**	**0.18**	**0.49**	**0.44**	**0.43**	**0.25**	**0.31**	**0.33**	**0.32**	**0.20**	—			
(17) TA	9.37 (1.37)	0.05	-0.08	*0.13*	0.03	0.10	**0.15**	0.02	*0.12*	0.04	0.10	*0.12*	0.11	0.08	**0.17**	*0.14*	**0.44**	—		
(18) LF	1.85 (.70)	-**0.21**	**0.38**	**0.21**	**0.32**	**0.45**	**0.30**	**0.23**	**0.35**	**0.35**	**0.35**	*0.14*	**0.32**	**0.20**	**0.22**	-0.09	**0.52**	*0.13*	—	
(19) AFI	11.57 (10.38)	**0.31**	0.06	0.12	0.03	0.02	*0.15*	0.03	*0.15*	0.09	0.01	0.06	*0.16*	0.07	0.02	**0.23**	**0.26**	**0.19**	0.06	—

*Bolded is p < 0.01, italicized is p < 0.05. N = 339. AFI, Activity Frequency Inventory; PPI-R, Psychopathic Personality Inventory-Revised; FD, Fearless Dominance; SCI, Self-centered Impulsivity; CH, Coldheartedness; AHPS, Authentic and Hubristic Pride Scales; AP, Authentic Pride; HP, Hubristic Pride; LSRP, Levenson “Self-report” Psychopathy Scale; F1, Factor 1; F2, Factor 2; CAB, Criminal and Analogous Behavior Scale; DPES, Dispositional Positive Emotionality Scale; RSES, Rosenberg Self-esteem Scale; MLQ, Multifactor Leadership Scale; TF, Transformational Leadership; TA, Transactional Leadership; LF, Laissez-faire leadership; GASP, Guilt and Shame Proneness Scale; NBE, Negative Behavior Evaluation; NSE, Negative Self Evaluation; GR, Guilt Response; SW, Shame Withdrawal*.

In contrast, AHPS Hubristic Pride was positively correlated with both PPI-R Fearless Dominance and Self-centered Impulsivity (*r*s were 0.21 and 0.42, respectively; see **Table 1**). At the subscale level, PPI-R Fearlessness and PPI-R Social Influence, but not PPI-R Stress Immunity, were significantly correlated with AHPS Hubristic Pride.

#### Guilt

Psychopathy measures manifested medium to strong negative correlations with GASP subscales that describe guilt (*r*s ranged from -0.16 to -0.56; see **Table 1**), although relations were significantly more pronounced for PPI-R Self-centered Impulsivity and PPI-R Coldheartedness than PPI-R Fearless Dominance (respectively, Steiger’s *Z*s were 4.45, *p* < 0.001 and 5.30, *p* < 0.001 for GASP Guilt-NBE, and 5.98, *p* < 0.001 and 5.26, *p* < 0.001 for GASP Guilt-Repair). Of the GASP subscales that describe shame, PPI-R Fearless Dominance was negatively correlated with both GASP Shame-NSE and GASP Shame-Withdraw; PPI-R Coldheartedness was strongly negatively correlated with GASP Shame-NSE and was not significantly correlated with GASP Shame-Withdraw (Steiger’s *Z* = -8.00, *p* < 0.001); PPI-R Self-centered Impulsivity was negatively correlated with GASP Shame-NSE and positively correlated with GASP Shame-Withdraw (Steiger’s *Z* = -9.35, *p* < 0.001).

#### Positive Parenting

Psychopathy higher-order dimensions were consistently negatively related to APQ Positive Parenting (*r*s = -0.16 to -0.29; see **Table 1**), except for PPI-R Fearless Dominance, which was positively related. Consistent with Lykken’s hypothesis, AHPS Authentic Pride, RSES, and DPES were positively associated with APQ Positive Parenting (*r*s from 0.37 to 0.39; see **Table 1**). There was no significant association between APQ Positive Parenting and AHPS Hubristic Pride.

#### Antisocial Behavior

As shown in **Table 1**, the antisocial behavior composite assessed by means of the CAB was significantly associated with PPI-R Fearless Dominance and Self-centered Impulsivity but not PPI-R Coldheartedness.

#### Prosocial Behavior: Heroism and Leadership

Consistent with previous findings, PPI-R Fearless Dominance and PPI-R Fearlessness (*r* = 0.26) manifested significant relations with the AFI, whereas PPI-R Self-centered Impulsivity and PPI-R Coldheartedness were not significantly correlated with the AFI (see **Table 1**). Further, the AFI was significantly related to MLQ Transformational Leadership and MLQ Transactional Leadership, but, also, to the CAB antisocial behavior composite (*r*s ranged from 0.19 to 0.26; see **Table 1**). Similarly, MLQ Transformational Leadership manifested significant positive correlations with PPI-R Fearless Dominance and PPI-R Coldheartedness, and significant negative correlations with PPI-R Self-centered Impulsivity.

### Specificity: The Role of Narcissism and General Personality Traits

#### Narcissism

Psychopathy dimensions demonstrated divergent patterns of correlations with the NPI’s three dimensions (see **Table 2**). PPI-R Fearless Dominance was more robustly correlated with NPI Leadership/Authority than were either PPI-R Self-centered Impulsivity or PPI-R Coldheartedness (Steiger’s *Z*s were 8.71, *p* < 0.001, and 8.88, *p* < 0.001, respectively). Similarly, PPI-R Fearless Dominance was more robustly correlated with NPI Grandiose Exhibitionism than were either PPI-R Self-centered Impulsivity (Steiger’s *Z* = 4.28, *p* < 0.001) or PPI-R Coldheartedness (Steiger’s *Z* = 4.56, *p* < 0.001). Conversely, relations between PPI-R Self-centered Impulsivity and PPI-R Coldheartedness, on the one hand, and NPI Entitlement/Exploitativeness, on the other, were significantly more pronounced than for PPI-R Fearless Dominance and NPI Entitlement/Exploitativeness (Steiger’s *Z*s = 3.57, *p* < 0.001, and 3.35, *p* < 0.001). These findings are consistent with Ackerman et al. (2011)’s conceptual and empirical description of their three-factor solution, whereby Leadership/Authority assesses largely adaptive aspects of personality, Grandiose Exhibitionism assesses mixed outcomes, and Entitlement/Exploitativeness assesses largely negative outcomes.

**Table 2:** Correlations between pride, psychopathy, narcissism, and general personality.

	NPI			HEXACO					
	L/A	GE	E/E	*H*	*E*	*X*	*A*	*C*	*O*
AHPS AP	**0.51**	**0.37**	0.00	**0.16**	**-0.20**	**0.77**	**0.31**	**0.37**	**0.17**
AHPS HP	**0.30**	**0.45**	**0.38**	**-0.30**	-0.11	0.07	**-0.20**	**-0.18**	-0.01
RSES	**0.39**	**0.23**	-0.11	**0.23**	**-0.21**	**0.74**	**0.32**	**0.37**	**0.20**
DPES	**0.55**	**0.36**	-0.01	**0.19**	**-0.22**	**0.79**	**0.29**	**0.36**	**0.25**
PPI-R FD	**0.72**	**0.55**	**0.24**	-0.05	**-0.50**	**0.76**	*0.14*	*0.12*	**0.23**
PPI-R SCI	**0.21**	**0.28**	**0.48**	**-0.49**	*-0.15*	**-0.24**	**-0.55**	**-0.54**	-0.06
PPIR CH	**0.24**	**0.279**	**0.45**	**-0.62**	**-0.61**	-0.02	**-0.27**	**-0.29**	**-0.30**

*N = 339. Bolded is p < 0.01, italicized is p < 0.05. NPI, Narcissistic Personality Inventory; L/A, Leadership/Authority; GE, Grandiose Exhibitionism; E/E, Entitlement/Exploitativeness; HEXACO, HEXACO Personality Inventory; H, Honesty/Humility; E, Emotionality; X, Extraversion; A, Agreeableness; C, Conscientiousness; O, Openness to experience; AHPS, Authentic and Hubristic Pride Scales; AP, Authentic Pride; HP, Hubristic Pride; RSES, Rosenberg Self-esteem Scale; DPES, Dispositional Positive Emotionality Scale; PPI-R, Psychopathic Personality Inventory-Revised; FD, Fearless Dominance; SCI, Self-centered Impulsivity; CH, Coldhearteness.*

AHPS Authentic Pride, RSES, and DPES were uniformly positively correlated with both NPI Leadership/Authority (*r*s ranged from 0.39 to 0.55) and NPI Grandiose Exhibitionism (*r*s ranged from 0.23 to 0.37) but none were significantly correlated with NPI Entitlement/Exploitativeness. AHPS Hubristic Pride manifested medium-to-strong positive correlations with all three NPI dimensions (*r*s ranged from 0.30 to 0.45).

#### HEXACO Personality Traits

See **Table 2** for interrelations between personality traits, as assessed by the HEXACO, and pride measures. Most notably, AHPS Authentic Pride, RSES, and DPES were all positively correlated with HEXACO Extraversion (r*s* ranged from 0.74 to 0.79), HEXACO Honesty/Humility (*r*s from 0.16 to 0.23), HEXACO Agreeableness (*r*s from 0.29 to 0.32), HEXACO Conscientiousness (*r*s from 0.36 to 0.37), and HEXACO Openness to experience (*r*s from 0.17 to 0.25), whereas AHPS Hubristic Pride was either negatively correlated—as was the case for HEXACO Honesty/Humility, HEXACO Agreeableness, and HEXACO Conscientiousness—or not significantly correlated with those same variables.

### Testing Lykken’s Hypothesis: Moderators of the Psychopathy-Social Behavior Link

Using the PROCESS macro (Hayes, 2017) in SPSS, we conducted a series of moderated multiple regression analyses to examine whether the relation between psychopathy (especially Fearless Dominance) and social behavior differed as a function of positive parenting and/or pride. We tested all possible combinations of moderation, 30 of which were hypothesis-driven, with all PPI-R higher-order dimensions, PPI-R Fearlessness, and both LSRP factors serving as the predictors; authentic and hubristic pride and positive parenting as the moderators; and antisocial and prosocial behavior, the latter by way of heroism and leadership style, as the external criteria. Given the large number of moderation analyses, we adopted a conservative p level of 0.005 to provide a reasonable balance between Type I and Type II error. Notable findings are reported below.

#### Antisocial Behavior

Contrary to expectations, APQ Positive Parenting, the DPES, and the RSES did not significantly moderate the relation between psychopathic traits and antisocial behavior. AHPS Authentic Pride moderated the relations between PPI-R Fearless Dominance and CAB antisocial behavior in a potentiating manner, such that the addition of the interaction term accounted for a small, non-significant, proportion of variance in the outcome [Δ*R^2^* = 0.040, *F*(1,315) = 3.94, *p* = 0.048]. Note, however, that the direction of this effect, which was potentiating, was opposite to that predicted. This finding held for PPI-R Coldheartedness (Δ*R^2^* = 0.027, *p* < 0.001), and LSRP F1 (Δ*R^2^* = 0.020, *p* = 0.013), although the latter finding, as with PPI-R Fearless Dominance, fell short of our more conservative significance threshold.

Again inconsistent with our predictions, AHPS Hubristic Pride moderated the relations between PPI-R Self-centered Impulsivity and CAB antisocial behavior, this time in a protective manner, such that the interaction term accounted for a significant proportion of variance in the outcome [Δ*R^2^* = 0.040, *F*(1,315) = 9.90, *p* = 0.002]. This finding suggests that AHPS Hubristic Pride may, in fact, protect against criminal and antisocial behaviors in individuals with elevated interpersonal-affective facets of psychopathy (e.g., lying, lack of remorse or guilt, low empathy, and callousness).

#### Prosocial Behavior

Results for prosocial behavior were more promising, but mixed. As predicted, both the DPES and the RSES significantly moderated the relation between PPI-R Fearless Dominance and MLQ Transformational Leadership in a potentiating manner, such that the interaction terms accounted for a significant increase in the variance of the outcome [DPES: Δ*R^2^* = 0.039, *F*(1,296) = 18.44, *p* < 0.001; RSES: Δ*R^2^* = 0.023, *F*(1,294) = 9.55, *p* = 0.002]. Further, DPES scores moderated the relation between PPI-R Fearless Dominance and MLQ Transactional Leadership in a potentiating manner [Δ*R^2^* = 0.025, *F*(1,296) = 7.79, *p* = 0.006], although the latter finding again fell short of our more conservative significance threshold. No other significant interaction effects were found across all analyses for relations between our predictor variables and prosocial behavior as measured by the MLQ and AFI.

## Discussion

Our investigation sought to evaluate Lykken’s (1995) conjecture that pride protects against antisocial behavior and perhaps promotes prosocial behavior in individuals with marked psychopathic traits, especially fearlessness. Further, Lykken contended that parents and other socializing agents could inculcate pride by imbuing pre-psychopathic children with a healthy self-concept. Taken together, our findings yielded a number of new and intriguing insights, but decidedly mixed support for the contention that pride moderates the relation between fearlessness and social behavior.

One of our central hypotheses—namely, that authentic pride would attenuate the risk of antisocial behaviors among individuals with high levels of fearless dominance—received minimal support. We found mixed support for the additional hypothesis that authentic pride would potentiate the association between fearless dominance and prosocial behavior. Consistent with our predictions, healthy pride moderated (potentiated) the relation between fearless dominance and adaptive leadership behaviors (i.e., transformational leadership). Contrary to our predictions, however, this relation did not extend to everyday heroism. Pending independent replication, these data provisionally indicate that authentic pride may be a partial shaping force in “successful” or adaptive psychopathy, a construct that shares considerable conceptual overlap with the archetypal “corporate” psychopath. Further, our findings raise the possibility that individuals high in both fearless dominance and authentic pride may be particularly successful leaders relative to other individuals with psychopathic traits. Prideful psychopaths who are successful leaders may strike an effective balance of bold interpersonal impact and intermittent prosocial behavior—appearing suave, self-assured, and daring to their followers while, critically, corroborating their self-assuredness with “good” behavior stemming from their contingent positive self-regard (i.e., authentic pride). Still, while our positive findings concerning leadership do bear some important implications, they should be interpreted with a measure of caution.

Unexpectedly, hubristic pride moderated the relation between self-centered impulsivity and antisocial behavior in a protective manner. We can envision two mutually exclusive explanations for this puzzling finding. The first is that it reflects Type I error. The second is substantive, although *post hoc* and conjectural. Perhaps psychopathic individuals who are willing to endorse items on the Hubristic Pride Scale with negative connotations such as “conceited” or “arrogant” are especially cognizant of social norms surrounding expressions of egotism. In turn, this awareness may reflect a degree of internalized socialization that protects against antisocial behaviors (Holbrook et al., 2014b).

Although not directly relevant to Lykken’s hypothesis, our findings also yielded several previously unreported associations that point to meaningful zero-order relations between psychopathy and pride, and highlight the importance of distinguishing both broad constructs at the subdimension level. For instance, fearless dominance and self-centered impulsivity yielded differential relations with authentic and hubristic pride. Fearless dominance was significantly associated with both subtypes of pride, although it manifested significantly larger associations with authentic pride, consistent with conceptualizations of the former as largely adaptive (Lilienfeld et al., 2012).

The finding that fearless dominance was associated with both forms of pride, suggesting that it is neither entirely adaptive nor maladaptive, can be situated within a broader debate surrounding the relevance of largely adaptive traits within conceptualizations of psychopathy. Some authors (e.g., Lynam and Miller, 2012) contend that fearless dominance is merely peripheral to psychopathy and that its near null associations with externalizing behavior and other maladaptive outcomes raise questions concerning its construct validity as a psychopathy subdimension. In contrast, others (e.g., Lilienfeld et al., 2012) contend that fearless dominance captures the superficially normal “mask” of healthy functioning (Cleckley, 1941) that is part-and-parcel of psychopathy. Of course, our findings regarding the correlations of fearless dominance with facets of pride by no means resolve this debate. At the same time, they suggest that fearless dominance, whatever its relevance to psychopathy, appears to display at least some maladaptive correlates, at least concerning arrogance and related elements of malignant pride and narcissism.

Further, self-centered impulsivity was significantly positively associated with hubristic pride, which is consistent with conceptualizations of both constructs as maladaptive (e.g., Carver et al., 2010; Edens and McDermott, 2010), and negatively associated with authentic pride. Taken together, the differential relations of fearless dominance and self-centered impulsivity, on the one hand, with authentic and hubristic pride, on the other, suggest that psychopathic individuals’ self-concept depends on their level of component traits, with individuals with elevated levels of fearless dominance reporting a healthy self-concept and individuals with high levels of self-centered impulsivity being disposed to hubris and malignant self-esteem.

Lastly, although Lykken’s model was the focus of our primary analyses, in subsidiary analyses, we used path analysis to examine an alternate model that did not incorporate moderation. This model posited that fearless dominance and positive parenting would contribute directly to authentic and/or hubristic pride, which, in turn, would partially or fully mediate the relation between fearless dominance and social behavior. Hence, nested iterations of three overidentified structural models with predictor (i.e., exogenous) variables PPI-R Fearless Dominance and APQ Positive Parenting, the proposed mediators of AHPS Authentic Pride and AHPS Hubristic Pride, and differing criteria (e.g., CAB Antisocial Behavior, MLQ Transformational Leadership, and AFI Heroism) were evaluated using Ωnyx 1.0-972 (von Oertzen et al., 2015) with maximum-likelihood estimation. The Supplementary Materials contain path diagrams of each model, relative magnitudes of path coefficients representing the direct and indirect relations of fearless dominance and positive parenting to the outcome variables when mediated by authentic and hubristic pride, and fit indices for full and reduced (i.e., with authentic and/or hubristic pride’s a and b paths fixed to zero) models. Assuming the posited causal ordering of our variables was sound, results suggested that (a) both fearless dominance and positive parenting mediate antisocial behavior by way of authentic pride (See Supplementary Figure 1); (b) authentic pride and hubristic pride completely mediate the relation between fearless dominance and transformational leadership (positively for authentic pride and negatively for hubristic pride) (See Supplementary Figure 4); and (c) pride appears to be relevant to psychopathy and social behavior given that models adjusted to elide either authentic or hubristic pride resulted in a significant decrement in fit. Nevertheless, given that these models were exploratory (not predicted) and based on cross-sectional data, they should be interpreted with caution pending replication in longitudinal studies.

Despite its strong basis in theoretically informed conjectures (Lykken, 1995), our study was characterized by several limitations. First, our exclusive reliance on self-report instruments renders our findings potentially subject to mono-method bias. Nevertheless, granting this limitation, self-reported psychopathy subdimensions displayed dramatically different relations with forms of pride, suggesting at least some substantive variance rising above method-covariance. Future research should incorporate informant reports of both psychopathy and pride in addition to self-report to provide stronger corroboration of the findings, especially because individuals with high levels of psychopathy and hubristic pride may be marked by blind spots reflecting a lack of insight (Lilienfeld and Fowler, 2006).

Second, our measure of parenting was retrospective and may have been biased by respondents’ current personality traits. For example, perhaps individuals who think highly of themselves attribute their high-esteem in part to their upbringing and thereby recall their parenting as especially positive. Alternatively, it may be that self-reports of pride dimensions and positive parenting are both influenced by a third variable, such as individual differences in positive emotionality. Future tests of Lykken’s (1995) hypothesis would benefit from longitudinal follow-ups of the statistical interaction between parenting styles and fearlessness among children.

Third, the construct validity of the Authentic and Hubristic Pride Scales is questionable. Holbrook et al. (2014a) offered theory- and data-driven challenges to certain facets of the Authentic/Hubristic Pride model, as well as to the construct validity of the Authentic and Hubristic Pride Scales. Specifically, they argued that AHPS Authentic Pride indexes both adaptive (i.e., achievement-oriented; authentic) and maladaptive (i.e., effort-oriented; hubristic) pride, whereas AHPS Hubristic Pride conflates hubristic pride with cognizance of social norms and/or an individual’s belief that they have engaged in socially proscribed pridefulness (c.f., Tracy and Robins, 2014). This suboptimal operationalization of our target constructs may have led us to commit what and Kimball (1957) and Kaiser (1960) (among others) called a “Type III error,” wherein researchers provide the right answer to the wrong question. Along with informant reports, future research should consider examining non-verbal pride behaviors (e.g., enlarged posture, arms on hips or raised above the head, head tilted backward), which appear across many or most cultures and may signal heightened standing or proficiency (Tracy et al., 2005; Tracy and Robins, 2008), dominance (Williams and DeSteno, 2009) and social attractiveness (Verbeke et al., 2004).

Fourth, we relied exclusively on an M-Turk sample to investigate these relations. Although community samples appear to be largely representative of the broader population (Miller et al., 2017), future research should examine the generalizability of our findings to other samples, particularly those marked by potentially high rates of both prosocial and antisocial behaviors, including corporate and political samples.

Fifth and finally, given that several of our findings were unexpected, or borne of exploratory analyses, a confirmatory, follow-up experiment may allow researchers to better understand the role of pride in shaping the behavioral expressions of psychopathic traits. For instance, future researchers could induce participants to experience either authentic or hubristic pride on a state basis (e.g. McFerran et al., 2014) and subsequently examine their self-reported or behaviorally measured prosocial and antisocial behaviors.

Another potential avenue for future research concerns recent scholarship demonstrating that, although certain psychopathic individuals may have relatively small, hypoactive amygdalae (Jones et al., 2009; Pardini et al., 2014) and exhibit reduced amygdala responses to fearful facial expressions (e.g., Dawel et al., 2012), “extreme” altruists (e.g., individuals who have donated organs to strangers) exhibit enhanced amygdala responsivity and sensitivity to fearful facial expressions (Marsh, 2016). Marsh et al. (2014) posited that these findings reflect a shared cognitive and/or neural mechanism underlying social behavior, such that antisocial and prosocial behavior occupy opposing points on a spectrum of behavior. Still, the degree to which Marsh and colleagues’ important work challenges Lykken’s low-fear hypothesis remains unclear. Fearlessness is especially relevant to imminent threat (Sylvers et al., 2011), whereas organ donation and other variants of extreme altruism are typically the products of lengthy deliberation. A paucity of fear may allow a heroic individual to charge into a burning building, but an organ donor’s fear of bodily harm may, with time, be outweighed by their empathy for the organ recipient^1^. Moreover, positive associations between antisocial behavior and heroic behavior were demonstrated in the current study as well as in several previous investigations in community and undergraduate samples (Smith et al., 2013), suggesting that certain forms of prosocial behavior (i.e., heroism) and criminality are not necessarily antithetical. Hence, the implications of Marsh and colleagues work on altruism and psychopathic individuals for moderated expression models of psychopathy (see Hall and Benning, 2006) is an important area of future research.

Our mixed results, along with these limitations and qualifications, notwithstanding, our findings are heuristically valuable in pointing to the largely neglected role of pride in understanding the correlates and potential behavioral manifestations of psychopathic traits. Given that pride may be an alternative venue to adequate socialization in individuals who are deficient in guilt, we strongly encourage psychopathy researchers to pay greater heed to this construct and its subtypes.

## Ethics Statement

This study was carried out in accordance with the recommendations of Emory University Institutional Review Board (IRB) Policies and Procedures, and the Emory IRB. The protocol was approved by the Emory University IRB. All subjects gave written informed consent in accordance with the Declaration of Helsinki.

## Author Contributions

AU and SL contributed the conception of the study. SL, AU, and AW provided the research design and methodological approach. AU and AW oversaw the collection of data. TC, SL, AU, and AW organized the dataset. TC and AW performed the statistical analysis and interpretation. TC wrote the first draft of the manuscript. TC, SL, and AU wrote sections of the manuscript. All authors contributed to manuscript revision, read, and approved the submitted version.

## Conflict of Interest Statement

The authors declare that the research was conducted in the absence of any commercial or financial relationships that could be construed as a potential conflict of interest.

## Footnotes

1 Notably, the HEXACO Personality Inventory includes an “Interstitial” scale known as HEXACO Altruism (versus Antagonism). The subscale, however, is largely a measure of sympathy, kindness, and soft-heartedness to others, and so is not directly relevant to our hypotheses concerning prosocial behavior. Exploratory analyses revealed that HEXACO Altruism was significantly negatively correlated with PPI-R Fearlessness (*r* = -0.28) and was not significantly correlated with PPI-R Fearless Dominance (*r* = -0.05). Further results concerning HEXACO Altruism are available from the corresponding author upon request.
