# Supplementary material for: Psychopathy and Pride: Testing Lykken’s Hypothesis Regarding the Implications of Fearlessness for Prosocial and Antisocial Behavior
Source: Front Psychol. 2018 Feb 20;9:185. doi: 10.3389/fpsyg.2018.00185 (PMC5827669; doi:10.3389/fpsyg.2018.00185)
Supplement: Supplementary file 1 [file Path_Analyses.docx]

Supplementary Material

Psychopathy and Pride: Testing Lykken’s Hypothesis Regarding the Implications of Fearlessness for Prosocial and Antisocial Behavior

Thomas H Costello^1*^, Ansley Unterberger^1^, Ashley L. Watts^1^, Scott O. Lilienfeld^1,2^

^1^Department of Psychology, Emory University, Atlanta, GA, USA

^2^University of Melbourne, Melbourne, Victoria, Australia

*** Correspondence:**Corresponding Author
thcoste@emory.edu

As stated in the paper, nested iterations of three overidentified structural models with differing outcome variables (e.g., CAB Antisocial Behavior, MLQ Transformational Leadership, and AFI Heroism) were evaluated using the Ωnyx 1.0-972 (von Oertzen, Brandmaier, & Tsang, 2015) with maximum likelihood estimation. Given the theory-driven nature of path analysis, we here opted only to examine a priori hypotheses that were judged to be conceptually robust. Namely, only hypotheses closely derived from Lykken’s theory concerning interrelations among fearless dominance, positive parenting, pride, and prosocial and antisocial behavior were tested.

**CAB/FD.** The first model examined the relations between PPI-R Fearless Dominance and CAB Antisocial Behavior, with APQ Positive Parenting, AHPS Authentic Pride, and AHPS Hubristic Pride as mediators of this relation; APQ Positive Parenting was also included as a mediator of AHPS Authentic Prides’ indirect effect on PPI-R Fearless Dominance and CAB Antisocial Behavior (see Supplemental Figure 1). This model demonstrated good fit (χ² (2) = 260.0, p < .001, RMSEA = .07, CFI = .99), although it accounted for only 9% of the variance in CAB Antisocial Behavior. To test the utility of pride in these models, we tested a series of nested models in which either the paths between (a) PPI-R Fearless Dominance to AHPS Authentic Pride and from AHPS Authentic Pride to CAB Antisocial Behavior (Supplemental Figure 2; χ² = 260.0, p < .001, RMSEA = .33, CFI = .41) and (b) PPI-R Fearless Dominance to AHPS Hubristic Pride and AHPS Hubristic Pride to CAB Antisocial Behavior (Supplemental Figure 3; χ² = 260.0, p < .001, RMSEA = .11, CFI = .94) were fixed to zero. Both of these model adjustments resulted in a decrement in fit, suggesting that pride is necessary to best account for relations among predictor and criterion variables in the full model.

**Transformational/FD**. Results of a similar path analysis, but with outcome variable MLQ Transformational Leadership rather than CAB Antisocial Behavior, are presented in Supplemental Figure 4. The full model showed acceptable fit, χ² (2) = 326.85, p < .001, RMSEA = .07, and CFI = .99, and accounted for 28% of the variance in MLQ Transformational Leadership. To test the utility of pride in this model, we tested a series of nested models in which either the paths between (a) PPI-R Fearless Dominance to AHPS Authentic Pride and from AHPS Authentic Pride to MLQ Transformational Leadership (Supplemental Figure 5; χ² (4) = 326.9, p < .001, RMSEA = .36, CFI = .45) and (b) PPI-R Fearless Dominance to AHPS Hubristic Pride and AHPS Hubristic Pride to CAB Antisocial Behavior (Supplemental Figure 6; χ² (4) = 326.9, p < .001, RMSEA = .16, CFI = .89) were fixed to zero. As was the case when CAB Antisocial Behavior was the outcome variable, both model adjustments resulted in a decrement in fit, suggesting that both authentic and hubristic pride are of utility in these models.

**AFI/FD.** Finally**,** a path analysis model with predictor variables PPI-R FD and APQ Positive Parenting, mediator variables AHPS Authentic Pride, AHPS Hubristic Pride, and criteria variable AFI Heroism (see Supplemental Figure 7) was tested, and demonstrated acceptable fit, χ² (2) = 255.6, p < .001, RMSEA < .07, CFI = .99, accounting for 12% of the variance in AFI Heroism. A nested model, with paths from PPI-R Fearless Dominance to AHPS Authentic Pride and from AHPS Authentic Pride to AFI Heroism fixed at 0 (Supplemental Figure 8), demonstrated poor fit, with χ² (4) = 255.6, p < .001, RMSEA = .33, CFI = .42; further, a model with paths from PPI-R Fearless Dominance to AHPS Hubristic Pride and from AHPS Hubristic Pride to AFI Heroism fixed at 0 was also relatively poorly fitting (Supplemental Figure 9), such that χ² = 255.69 (4), p < .001, RMSEA = .11, CFI = .93.

Supplemental Figure 1. *Results of the path analysis for Fearless Dominance, Positive Parenting, Authentic and Hubristic Pride, and Antisocial Behavior.*


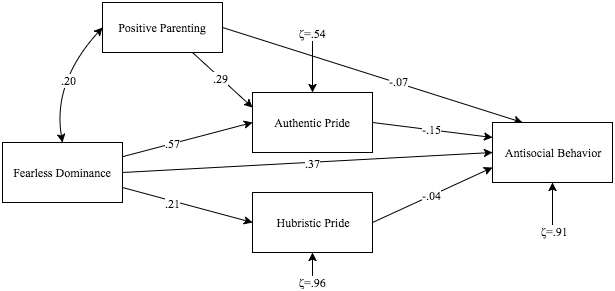


*Note.* Standardized coefficients are presented.

Supplemental Figure 2. *Results of the path analysis for Fearless Dominance, Positive Parenting, Hubristic Pride, and Antisocial Behavior.*


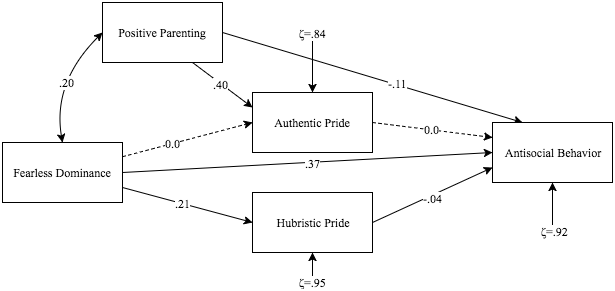


*Note*. Standardized coefficients are presented. Dotted lines indicate that a path is fixed at zero.

Supplemental Figure 3. *Results of the path analysis for Fearless Dominance, Positive Parenting, Authentic Pride, and Antisocial Behavior.*


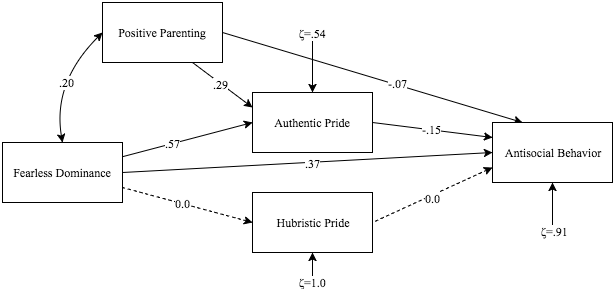


*Note*. Standardized coefficients are presented. Dotted lines indicate that a path is fixed at zero.

Supplemental Figure 4. *Results of the path analysis for Fearless Dominance, Positive Parenting, Authentic and Hubristic Pride, and Transformational Leadership.*


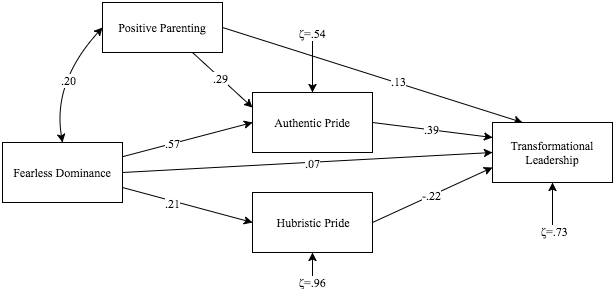


*Note*. Standardized coefficients are presented.

Supplemental Figure 5. *Results of the path analysis for Fearless Dominance, Positive Parenting, Hubristic Pride, and Transformational Leadership.*


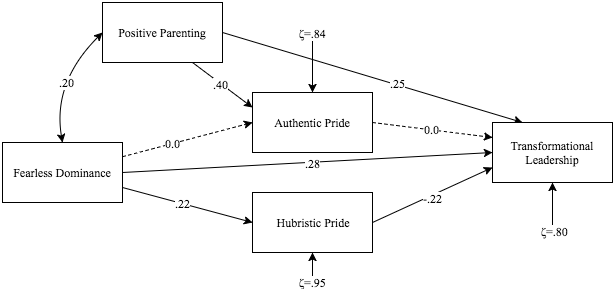


*Note*. Standardized coefficients are presented. Dotted lines indicate that a path is fixed at zero.

Supplemental Figure 6. *Results of the path analysis for Fearless Dominance, Positive Parenting, Authentic Pride, and Transformational Leadership.*


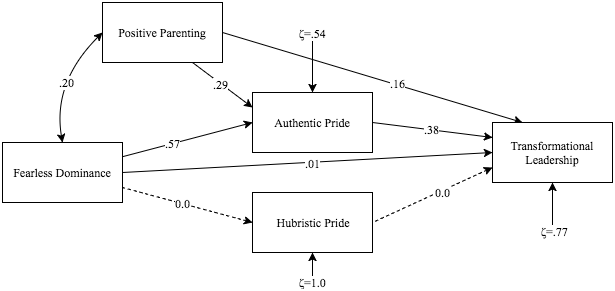


*Note*. Standardized coefficients are presented. Dotted lines indicate that a path is fixed at zero.

Supplemental Figure 7. *Results of the path analysis for Fearless Dominance, Positive Parenting, Authentic and Hubristic Pride, and Heroism.*


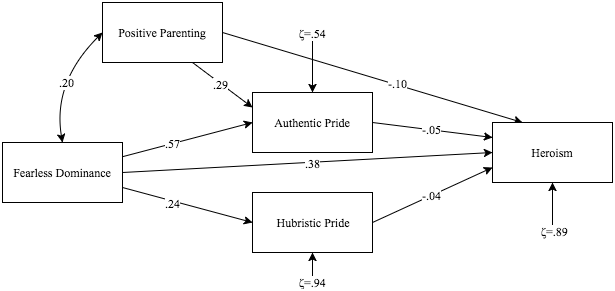


*Note*. Standardized coefficients are presented.

Supplemental Figure 8. *Results of the path analysis for Fearless Dominance, Positive Parenting, Hubristic Pride, and Heroism.*


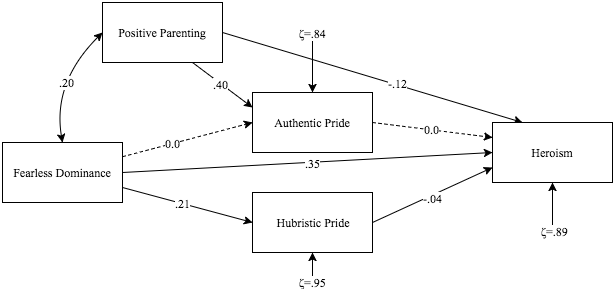


*Note*. Standardized coefficients are presented. Dotted lines indicate that a path is fixed at zero.

Supplemental Figure 9. *Results of the path analysis for Fearless Dominance, Positive Parenting, Authentic Pride, and Heroism.*


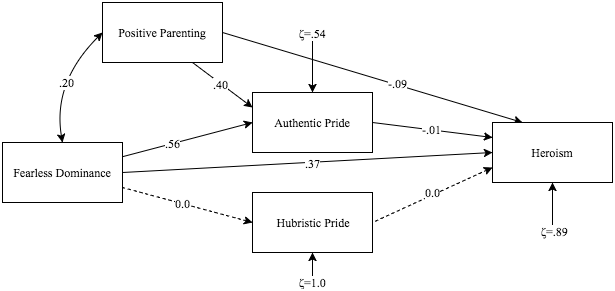


*Note*. Standardized coefficients are presented. Dotted lines indicate that a path is fixed at zero.
